# Umbilical Cord Serum Erythropoietin Levels and Maternal Smoking in Pregnancy

**DOI:** 10.1100/2012/420763

**Published:** 2012-05-01

**Authors:** Soner Sazak, Sinan Mahir Kayıran, Yahya Paksoy

**Affiliations:** ^1^Department of Pediatrics, Vakif Gureba Teaching and Training Hospital, Istanbul, Turkey; ^2^Department of Pediatrics, American Hospital, Istanbul, Turkey

## Abstract

*Objective*. To evaluate the effect of maternal smoking during pregnancy on levels of umbilical cord erythropoietin. *Methods*. Erythropoietin levels were measured in umbilical cord sera of 60 newborns who were delivered vaginally at term. There were 20 (33%) smoking and 40 (67%) nonsmoking mothers. *Results*. Mean cord serum erythropoietin levels were significantly lower in the nonsmokers (nonsmokers, 24 ± 9 IU/L; smokers, 61 ± 46 IU/L; *P* < .001). There was a significant positive correlation between the number of cigarettes smoked per day and cord serum erythropoietin levels (*r*, 0.58; *P* ≤ .05). *Conclusions*. Smoking during pregnancy is associated with increased levels of umbilical cord erythropoietin at birth. This may indicate a risk of fetal hypoxia and growth restriction. Education and encouragement of cessation of smoking during pregnancy are important to avoid associated fetal and maternal morbidity and mortality.

## 1. Introduction

Maternal smoking during pregnancy causes important metabolic and biochemical changes and adaptive responses in the fetus and mother, resulting in an increased incidence of maternal and fetal complications such as intrauterine growth retardation and decreased fetal weight and size [1–4]. The effects of smoking are dose dependent, and the prevalence of complications is increased with increased duration and amount of smoking [1–4]. The effect of smoking is independent of race, number of parities, socioeconomic status, and weight. Other complications that may increase in frequency with maternal smoking during pregnancy include fetal death and stillbirth, abortion, spontaneous abortion, ectopic pregnancy, premature birth, premature rupture of membranes, placental abruption, placenta previa, low birth weight, and low Apgar score [1–4].

Erythropoietin is a glycoprotein hormone involved in the regulation of erythropoiesis during fetal and adult life. The only known stimulus for production of erythropoietin is decreased partial oxygen pressure [1]. Hypoxia stimulates the production of erythropoietin in the fetal liver and adult kidney [1, 5–7]. Erythropoietin does not cross the placenta, and umbilical cord blood erythropoietin is of fetal origin. Increased umbilical cord erythropoietin levels are evidence of chronic fetal hypoxia [8, 9] and have been associated with fetal morbidity and mortality [1, 2, 4, 10, 11]. Elevated plasma erythropoietin occurs in pregnancies complicated by fetal growth retardation, meconium, diabetes, preeclampsia, isoimmunization, and smoking [1].

This study was undertaken to investigate the effect of maternal smoking on umbilical cord plasma erythropoietin levels as a measure of chronic intrauterine hypoxia. We hypothesized that maternal smoking may cause fetal hypoxia which may cause elevated erythropoietin levels, that is, having a compansatuar effect on blood gas oxygenization values.

## 2. Methods

This study was a prospective study performed on 60 neonates between November 2009 and April 2010 in a tertiary care center. History of maternal smoking was present with 20 of these neonates. The nonsmoking and smoking mothers had similar mean age (Table 1), and smoking mothers consumed an average of 9 ± 3 cigarettes per day for 6 ± 2 months during the pregnancy. The criteria for inclusion in the study included (1) uncomplicated pregnancy; (2) absence of history of diabetes mellitus, hypertension, infection, or any medication during pregnancy; (3) vaginal delivery; (4) minimum Apgar score of 8 at 1st and 5th minutes; (5) clear amniotic fluid; (6) gestational age of 37 to 42 weeks according to the last day of menstruation and ultrasonographic evaluation; (7) weight compatible with gestational age (between 10th and 90th percentiles); (8) smoked prior to pregnancy and continued smoking during pregnancy. The nonsmoking group were not exposed to secondary smoking. There were no multiple births. Written informed consent was obtained from the mother. This study was approved by the local ethical committee and conducted according to the Declaration of Helsinki.

 After delivery, a segment of the umbilical cord was clamped and venous cord blood samples were immediately transferred into tubes containing heparin. The serum was separated by centrifugation (4000 rpm for 5 minutes) and stored (−20°C) for 1 month. Erythropoietin levels were measured in duplicate using a chemiluminescent enzyme immunoassay (DPC Immulite EPO kit, Siemens Medical Solutions Diagnostics, Los Angeles, CA).

### 2.1. Data Analysis

All the statistical analyses were performed with SPSS 17.0 for Windows (SPSS Inc., Chicago, IL).

Average results for the nonsmoking and smoking groups were compared by using Mann-Whitney rank sum test, and correlations were evaluated with regression analysis. Data were reported as mean ±SE. Statistically significant differences were defined by *P* ≤ .05.

## 3. Results

The nonsmoking and smoking mothers had similar average age, number of parities, and duration of gestation, and the neonates from nonsmoking and smoking mothers had similar birth weight and Apgar scores (Table 1). Neonates born from nonsmoking and smoking mothers had similar average umbilical cord blood gas values, but neonates born from smoking mothers had significantly greater cord serum erythropoietin levels than nonsmoking mothers (Table 1). A positive correlation was noted between cord erythropoietin level and average daily consumption of cigarettes (*r*, 0.58; *P* ≤ .05); no correlation was observed between erythropoietin level and average duration of smoking during pregnancy (*r*, −0.03; NS).

## 4. Discussion

The finding of elevated cord erythropoietin levels in neonates born from smoking mothers (Table 1) is consistent with the hypothesis that maternal smoking may cause fetal hypoxia and potential fetal complications [1, 2, 4, 5]. We think that normal arterial oxygen values are because of the compansatuar effect of the erythropoietin.

In 3 recent studies, the correlation between fetal hypoxia and umbilical cord erythropoietin levels was well established, but confounding variables were present [2, 12, 13]. In 1 study, neonates that were small for gestational age were included in the smoking mother group [12]. Another study included infants that were small for gestational age, and the effect of growth retardation on erythropoietin levels may have affected the results [13]. In another study with neonates that were small for gestational age, erythropoietin levels were higher in neonates born to mothers with a history of smoking, and the number of cigarettes smoked daily was correlated with erythropoietin levels [2]. In the present study, we included neonates that had body weight appropriate for gestational age and who had not been exposed to any adverse events, other than smoking, that may have caused hypoxia. Therefore, our results may reflect the effect of smoking on erythropoietin levels more directly than previous publications.

In the present study, the higher erythropoietin levels from neonates born to smoking mothers and the correlation between the amount of cigarette consumption and erythropoietin levels suggest that smoking has a harmful effect in terms of chronic fetal hypoxia and this hazardous effect of smoking is dose dependent. These findings are consistent with results of previous studies [1, 2] and extend the available information about maternal smoking and umbilical cord erythropoietin levels. Future studies could improve these data by measuring markers of smoking in the maternal blood.

Fetal hypoxia, which causes erythropoietin synthesis, may be caused by several mechanisms. Nicotine acts on the cardiovascular system by causing the release of catecholamines into the maternal circulation, resulting in tachycardia, peripheral vasoconstriction, and reduction of placental blood flow that may cause a poor nutritional and oxygenation status for the fetus. Cotinine, a metabolite of nicotine, increases the vasoconstrictive action of prostaglandin E2, and the accumulation of cotinine in the fetal bloodstream may contribute to premature labor and spontaneous abortion among smokers. Furthermore, carbon monoxide produced by cigarettes has strong affinity towards fetal hemoglobin, resulting in hypoxia. A recent report showed a positive correlation between the number of cigarettes smoked per day and the absolute nucleated red blood cell count, another marker of chronic hypoxia, and this also may explain the higher hemoglobin concentrations reported in fetuses of smokers [5].

Suboptimal fetal oxygenation may cause several perinatal complications in fetuses of women who smoke. Carbon monoxide may interfere with tissue oxygenation by decreasing the blood oxygen transportation capacity and shifting the oxyhemoglobin saturation curve to the left, resulting in hypoxemia and associated growth restriction. Smokers also have deficiencies of some nutrients such as zinc, carotene, and cholesterol. Furthermore, cigarette smoke may be inhaled through the nasal mucosa of infants and affect the growth of infants born to smokers [3, 11]. Further studies may explain the mechanisms by which chronic fetal hypoxia contributes to the pathogenesis of associated disorders such as intrauterine growth retardation, spontaneous abortion, placental abruption, placenta previa, premature birth, perinatal mortality, congenital anomalies, malignancies, minimal brain dysfunction, hyperkinesia, and sudden infant death syndrome [3].

The risk of perinatal and obstetric problems is likely related to the number of cigarettes smoked daily and the trimester of pregnancy with the highest exposure. The fetus gains most weight during the second half of pregnancy. The fetus is not just a passive smoker inhaling cigarette smoke involuntarily in an open environment, but it is highly vulnerable and susceptible to the risk of developmental disorders. When a mother smokes, she exposes her fetus to the components of cigarette smoke crossing the placenta and to alterations in oxygen transport, placental metabolism, and maternal metabolism secondary to smoking [1, 5, 10].

Limitations of the present study include that our study had small group of pregnants and did not compare the different amounts of cigarette consumption.

 Nevertheless, the findings of the present study support the practice of encouraging pregnant women to quit smoking. Furthermore, pregnant women who smoke should be monitored closely for signs of uteroplacental insufficiency such as fetal growth restriction.

## Figures and Tables

**Table 1:** Relation between maternal smoking and neonatal characteristics.*

	Nonsmokers	Smokers	*P* ≤ ^†^
Number of subjects	40	20	
Maternal age (y)	27 ± 5	25 ± 4	NS
Number of parities	2 ± 1	1.7 ± 0.8	NS
Duration of gestation (wk)	40 ± 1	40 ± 1	NS
Birth weight (kg)	3.4 ± 0.4	3.3 ± 0.3	NS
Sex of neonate			
Male	23 (57%)	10 (50%)	NS
Female	17 (43%)	10 (50%)	
Apgar score			
1st minute	8.3 ± 0.4	8.4 ± 0.4	NS
5th minute	9.5 ± 0.5	9.4 ± 0.5	
Cord blood			
pH	7.32 ± 0.07	7.3 ± 0.6	NS
PO_2_ (mm Hg)	25 ± 7	22 ± 3	NS
PCO_2_ (mm Hg)	44 ± 8	48 ± 10	NS
Erythropoietin (IU/L)	24 ± 9	61 ± 46	.001

*Data reported as mean ±SE or number (percent).

^†^NS, not significant (*P* > .05).
